# Indole Inhibits IncP-1 Conjugation System Mainly Through Promoting *korA* and *korB* Expression

**DOI:** 10.3389/fmicb.2021.628133

**Published:** 2021-03-19

**Authors:** Rui Xiong, Yuyang Liu, Jieying Pu, Jianping Liu, Dexiang Zheng, Jianming Zeng, Cha Chen, Yang Lu, Bin Huang

**Affiliations:** ^1^Department of Laboratory Medicine, The First Affiliated Hospital of Sun Yat-sen University, Guangzhou, China; ^2^Department of Laboratory Medicine, The Second Affiliated Hospital of Guangzhou University of Traditional Chinese Medicine, Guangzhou, China

**Keywords:** indole, conjugation, *E. coli*, antibiotic resistance, ciprofloxacin

## Abstract

Indole works as an interspecies signal molecule to regulate multiple physiological activities, like antibiotic resistance, acid resistance, and virulence. However, the effect of indole on conjugation is unknown. Here, with *Escherichia coli SM10*λπ as a donor strain that carries a chromosomally integrated conjugative RP4 plasmid, we explored the effect of indole on conjugation of a mobilizable pUCP24T plasmid imparting gentamycin resistance. The results showed that exogenous indole treatment inhibited conjugative transfer of pUCP24T from *SM10*λπ to recipient strains, *Pseudomonas aeruginosa PAO1* and *E. coli EC600*. Furthermore, raising endogenous indole production through overexpression of TnaA, a tryptophanase, in *SM10*λπ significantly inhibited both *SM10*λπ-*PAO1* and *SM10*λπ-*EC600* conjugation, whereas deficiency of *tnaA* reversed the phenotype. Subsequent mechanistic studies revealed that exogenous indole significantly inhibited the expression of mating pair formation gene (*trbB*) and the DNA transfer and replication gene (*trfA*), mainly due to the promotion of regulatory genes (*korA* and *korB*), and the result was confirmed in *tnaA* knockout and overexpression strains. Additionally, we found that both extracellular indole production and *tnaA* expression of *SM10*λπ were downregulated by ciprofloxacin (CIP). Intriguingly, one-eighth minimum inhibitory concentration of CIP treatment clearly facilitated both *SM10*λπ-*PAO1* and *SM10*λπ-*EC600* conjugation, and indole inhibited CIP-induced conjugation frequency. These data suggest that indole may play a negative role in the process of CIP-induced conjugation. This is the first study to reveal the biological function of indole-inhibiting conjugation and its role in CIP-induced conjugation, which may be developed into a new way of controlling the spread of antibiotic resistance.

## Introduction

Conjugation achieves the transmission of bacterial genetic materials, especially plasmids that carry antibiotic resistance genes, in a unidirectional manner from a donor cell to a recipient cell (Waksman, 2019), mediating the process of horizontal gene transfer (HGT) and inducing the spread of antibiotic resistance. It requires a type IV secretion system (T4SS), which is encoded by Mating pair formation (Mpf) genes to form the conjugative pore, and DNA transfer and replication (Dtr) genes-encoded relaxosome composed of the relaxase, which nicks at the origin of transfer (oriT) and other auxiliary proteins (Schröder and Lanka, 2005; De La Cruz et al., 2010; Koraimann and Wagner, 2014). Conjugative plasmids, such as the well-studied IncP-1α plasmid RP4, generally carry all the structural genetic elements required to perform transfer (an *oriT* site, Dtr, and Mpf genes) (Pansegrau et al., 1990; Frost et al., 2005; Carballeira et al., 2014). Specifically, expression of plasmid RP4 conjugation genes is regulated by KorA, KorB, and TrbA, which act by binding to conserved nucleotide sequences localized in replication and maintenance regions, as well as transfer-essential Tral and Tra2 regions (Balzer et al., 1992; Zatyka et al., 1994; Jagura-Burdzy and Thomas, 1997). In contrast, mobilizable plasmids carry partial genetic information necessary for transfer (an oriT region and Dtr), and so usually there is a need to leverage the corresponding conjugative apparatus of co-resident self-transmissible plasmids to effect transfer (Francia et al., 2004).

The process of conjugation can be affected and regulated by many factors, summed up from two perspectives: (i) the internal environment involved in the interactions between the different genetic elements and (ii) the external environment including antibiotics, metals, carbon compounds, and quorum sensing (QS) (Banuelos-Vazquez et al., 2017). Specifically, the sub-minimal inhibitory concentration (sub-MIC) of antibiotics, such as gentamicin (Gm), sulfamethoxazole, tetracycline, and ciprofloxacin (CIP), increases the frequency of conjugation (Jutkina et al., 2016, 2018; Shun-Mei et al., 2018). The SOS response was revealed to promote horizontal dissemination of antibiotic resistance genes (Beaber et al., 2004). As for the effects of QS, our previous study has showed that the QS system signal molecules N-acyl homoserine lactones (AHLs) of *Pseudomonas aeruginosa* inhibit conjugation by activating SdiA in *Escherichia coli* (Lu et al., 2017b). Because indole is a signal molecule like QS, we were intrigued to explore whether they may play a role in conjugation.

Indole is used as a biochemical identification index of bacteria, whose production is controlled by the tryptophanase operon, including a promoter, a regulatory gene *tnaC*, and two structural genes *tnaA* and *tnaB* encoding tryptophanase and tryptophan permease, respectively (Yanofsky et al., 1991). However, a growing number of studies elucidate the function of indole as a signal molecule: (i) indole is a plateauing signal molecule (Kobayashi et al., 2006) which acts extracellularly to activate genes of metabolic enzymes in a concentration-dependent manner (Wang et al., 2001) and (ii) indole increases drug resistance by inducing intrinsic xenobiotic exporter genes in *E. coli* (Hirakawa et al., 2005). But there are no reports about indole working as a signal molecule to regulate conjugation.

The biosynthesis of indole is closely related to many environmental factors, such as cell density, carbon source, temperature, pH, and antibiotics. In terms of the latter, *E*. *coli* produced a higher level of extracellular indole in the presence of ampicillin and kanamycin (Han et al., 2011). We have previously disclosed the promoting effect of CIP on conjugation (Shun-Mei et al., 2018), but the underlying mechanism, especially whether indole was involved in this process, was not explored. In this study, we aimed to determine if indole affects conjugation, if it further participates in the process of antibiotics-induced conjugation.

## Results

### Inhibition of Conjugation by Indole

**FIGURE 1:**
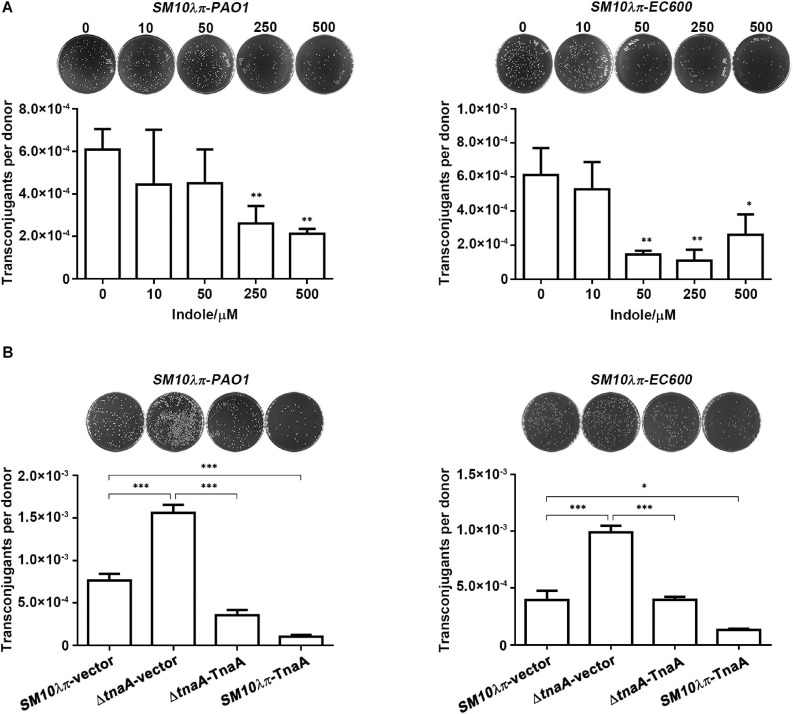
Indole inhibited *SM10*λπ-*PAO1* and *SM10*λπ-*EC600* conjugation. **(A)** Effect of exogenous indole on *SM10*λπ-*PAO1* and *SM10*λπ-*EC600* conjugation. Donor *SM10*λπ and recipient *PAO1* or *EC600* cells (1 × 10^7^ CFU/ml each) were mated in the presence of indicated concentrations of indole at 37°C for 6 h. **(B)** Effect of endogenous indole on *SM10*λπ-*PAO1* and *SM10*λπ-*EC600* conjugation. *SM10*λπ wild-type strain (*SM10*λπ-vector), *tnaA*-deficient strain (Δ*tnaA*-vector), TnaA overexpression strain in wild-type (*SM10*λπ-TnaA), and *tnaA* mutation (Δ*tnaA*-TnaA) were mated with *PAO1* or *EC600* cells (1 × 10^7^ CFU/ml each), respectively, at 37°C for 6 h. Values are means ± SEMs from at least three independent experiments; ^∗^*P* < 0.05; ^∗∗^*P* < 0.01; ^∗∗∗^*P* < 0.001.

To elucidate the effect of indole on both intraspecies and interspecies plasmid transfer, *E. coli SM10λπ*-*PAO1* and *SM10*λπ-*EC600* conjugational models established in our previous study were taken into application (Lu et al., 2017b). We first determined the appropriate concentration of exogenous indole that might not threaten the growth of *SM10*λπ, *PAO1*, and *EC600*, since it has been reported that bacterial conjugational efficiency is closely related to the growth state of bacteria (Schuurmans et al., 2014). As shown in Supplementary Figure 1, the growth of *SM10*λπ, *PAO1*, and *EC600* was only trivially affected by indole even at concentration as high as 500 μM. Then the appropriate concentrations (10, 50, 250, and 500 μM) of exogenous indole were used to treat *SM10*λπ-*PAO1* and *SM10*λπ-*EC600* conjugational models. The results showed that 250 μM indole was needed to start inhibiting *SM10*λπ-*PAO1* conjugation, while 50 μM indole was sufficient to inhibit *SM10*λπ-*EC600* conjugation. Both conjugation models were significantly depressed by indole in a dose-dependent manner (Figure 1A), although in the *SM10*λπ-*EC600* conjugation model, the conjugation frequency of 500 μM indole treatment group was higher than that of 250 μM indole treatment group, which may be interpreted as high concentration indole, like antibiotics, increases the frequency of conjugation by promoting the growth of transconjugants (Lopatkin et al., 2016).

To validate the role of endogenous indole in conjugation, we subsequently constructed *tnaA* gene knockout and overexpression strains in *SM10*λπ, as tryptophanase encoded by *tnaA* hydrolyzes tryptophan to create indole, pyruvate, and ammonia (Newton et al., 1965; Deeley and Yanofsky, 1981). Naturally, we can barely detect indole and *tnaA* expression in the *tnaA*-deficient strain (Δ*tnaA*-vector), while overexpression of TnaA in wild-type (*SM10*λπ-TnaA) and the *tnaA* mutation (Δ*tnaA*-TnaA) strain both delivered much higher levels of indole and *tnaA* expression (Supplementary Figure 2). We next examined the conjugation frequency by using these constructed strains as donor cells. Compared to the wild-type strain, deficiency of *tnaA* in *SM10*λπ significantly enhanced both *SM10*λπ-*PAO1* and *SM10*λπ-*EC600* conjugation, whereas introducing a plasmid of TnaA overexpression reversed the phenotype. In addition, introducing a TnaA overexpression plasmid into the *SM10*λπ wild-type strain also remarkably inhibited conjugation frequency in both conjugation models (Figure 1B).

Collectively, these data suggest that indole plays a role in inhibiting conjugation.

### Effects of Indole on the Expression of Conjugation Genes and Global Regulatory Genes

**FIGURE 2:**
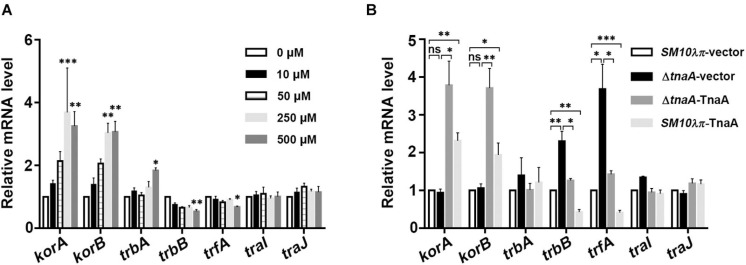
The mRNA expression level of conjugation-associated genes. **(A)** Effect of exogenous indole on the expression levels of conjugation genes (*trbB*, *trfA*, *traI*, and *traJ*) and global regulatory genes (*korA*, *korB*, and *trbA*). *SM10*λπ (1 × 10^7^ CFU/ml) was treated with different concentrations of indole at 37°C for 3 h, followed by real-time PCR analysis. **(B)** Effect of endogenous indole on the expression of the aforementioned conjugation-associated genes. The indicated *SM10*λπ strains were mated (1 × 10^7^ CFU/ml) at 37°C for 3 h, followed by real-time PCR analysis. The *rpoD* gene of *SM10*λπ was used as an internal control. Values are means ± SEMs from at least three independent experiments; ^∗^*P* < 0.05; ^∗∗^*P* < 0.01; ^∗∗∗^*P* < 0.001; ns, not significant.

To further explore the molecular mechanisms underlying the regulatory function of indole on conjugation, we measured the mRNA expression of the major global regulatory genes (*korA*, *korB*, and *trbA*) and conjugation genes, including Mpf gene (*trbB*), the Dtr gene (*trfA*), gene which encodes conjugative transfer relaxase (*traI*), and gene which activates *tra* gene expression (*traJ*). The results showed that the mRNA levels of *korA* and *korB* were unambiguously increased along with the addition of 250 μM indole (Figure 2A), and the promotion was observed as well in strains of overexpression of TnaA (Δ*tnaA*-TnaA and *SM10*λπ-TnaA) (Figure 2B), but no expression change of *korA* and *korB* was observed in *tnaA*-deficient strain. On the other hand, *trbA* was up-regulated slightly even when indole concentration was up to 500 μM (Figure 2A), and the mRNA expression level of *trbA* showed no significant difference in both knockout and overexpression strains (Figure 2B).

Consequently, the mRNA expression levels of *trbB* and *trfA* decreased significantly with increasing indole concentrations (Figure 2A). Additionally, compared to the wild-type strain, deficiency of *tnaA* in *SM10*λπ significantly enhanced both the expression of *trbB* and *trfA*, whereas introducing a plasmid of TnaA overexpression reversed it, and introducing a TnaA overexpression plasmid into the *SM10*λπ wild-type strain also remarkably inhibited the mRNA expression levels of *trbB* and *trfA* (Figure 2B). However, the expression of *traI* and *traJ* was barely affected by exogenous indole treatment (Figure 2A) and showed no significant difference in knockout and overexpression strains (Figure 2B).

Taken together, these results suggest that indole may inhibit conjugation mainly through regulating the expression of *korA* and *korB*, further regulating the expression of *trbB* and *trfA*.

### Indole Plays a Negative Role in the Process of CIP-Induced Conjugation

**FIGURE 3:**
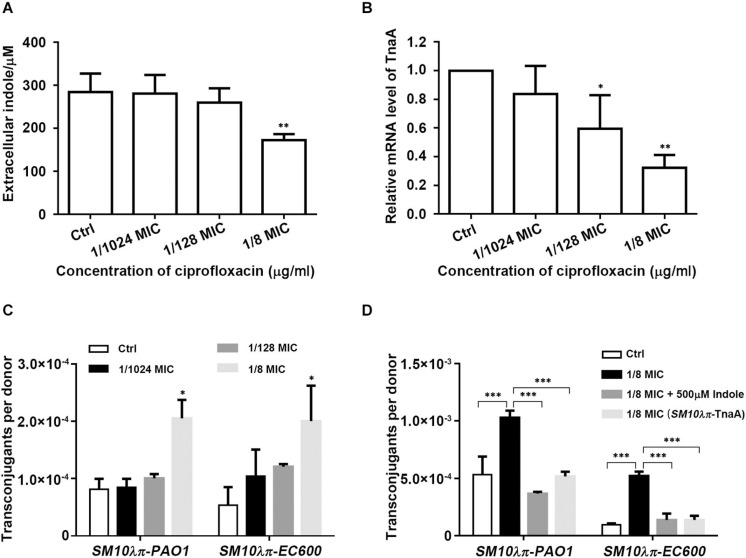
Indole inhibited CIP-induced conjugation. **(A)** Effect of CIP on indole production. *SM10*λπ was treated with different sub-MICs of CIP at 37°C for 5 h, followed by measurement of extracellular indole. **(B)** Effect of CIP on *tnaA* expression. *SM10*λπ was treated with different sub-MICs of CIP at 37°C for 4 h, followed by real-time PCR analysis of *tnaA* expression. **(C)** Effect of CIP on *SM10*λπ-*PAO1* and *SM10*λπ-*EC600* conjugation. *SM10*λπ was treated with different sub-MICs of CIP at 37°C for 8 h and then mated with *PAO1* or *EC600* cells at 37°C for 6 h. **(D)** Indole inhibited CIP-induced conjugation frequency. *SM10*λπ was treated with 1/8 MIC of CIP at 37°C for 8 h and then mated with *PAO1* or *EC600* cells in the presence of 500 μM of indole at 37°C for 6 h; meanwhile, overexpression of TnaA in wild-type (*SM10*λπ-TnaA) was treated with 1/8 MIC of CIP at 37°C for 8 h and then mated with *PAO1* or *EC600* cells at 37°C for 6 h. Values are means ± SEMs from at least three independent experiments; ^∗^*P* < 0.05; ^∗∗^*P* < 0.01; ^∗∗∗^*P* < 0.001.

We have previously reported that sub-MIC of CIP promoted *SM10*λπ-*PAO1* conjugation, but the underlying mechanism was not explored. Here, we measured extracellular indole production and *tnaA* expression of *SM10*λπ after cultivation in the presence of CIP at sub-inhibitory concentrations (1/8 MIC, 1/128 MIC, and 1/1024 MIC) and found that 1/8 MIC CIP significantly repressed indole production (Figure 3A) and *tnaA* expression (Figure 3B). It seems that indole may play a role in CIP-induced conjugation, which needs further confirmation. We next added the *SM10*λπ-*EC600* conjugation model and further confirmed that treatment with 1/8 MIC of CIP clearly enhanced conjugation frequency in both *SM10*λπ-*PAO1* and *SM10*λπ-*EC600* models (Figure 3C). Exogenous indole was added after conjugation system was treated with CIP, or overexpression of TnaA, when wild-type (*SM10*λπ-TnaA) was used as donor cell in CIP-treated conjugation models. It turned out that indole inhibited CIP-induced conjugation frequency in both conjugation models (Figure 3D). These data suggested that indole may play a negative role in the process of CIP-induced conjugation.

## Discussion

The physiological functions of indole, like maintaining plasmid stability, affecting biofilm formation, and antibiotic resistance, have been the subject of active investigation (Chant and Summers, 2007; Nishino et al., 2008; Kuczynska-Wisnik et al., 2010). Here, we explored the role of indole in the regulation of intraspecies and interspecies conjugation. Both exogenous indole treatment and gain- and loss-of-function studies showed the inhibitory effect of indole on *SM10*λπ-*PAO1* and *SM10*λπ-*EC600* conjugation. Compared to most research into the conjugational regulatory mechanism that has focused on self-transmissible elements (Frost et al., 2005), this study and our previous work (Lu et al., 2017b) revealed that chemical signal molecules, namely, indole and AHLs that are produced by donor and recipient cells, respectively, may inhibit conjugation.

Mpf gene, *trbB*, is responsible for the development of the conjugative pore, and the Dtr gene, *trfA*, encodes a *trans*-acting product essential for vegetative plasmid replication (Smith and Thomas, 1984; Zatyka et al., 1997). Global regulatory genes *korA* and *korB* both exert roles in repression of *trfA* expression, and *trbA* and *korB* expression severely represses the *trbB* promoter (Schreiner et al., 1985; Theophilus et al., 1985; Zatyka et al., 2001). We found that exogenous indole treatment significantly up-regulated the mRNA expression levels of *korA* and *korB*, which in turn inhibited the expression of *trbB* and *trfA* genes, thereby decreasing the conjugation frequency. Additionally, the results of the aforementioned genes’ expression in *tnaA* knockout and overexpression strains further confirmed the regulatory mechanism of indole on conjugation. However, it is worth noting that although *korA* and *korB* expression did not decrease in *tnaA*-deficient strain, the expression of *trbB* and *trfA* increased. It may be that the expression of *trbB* and *trfA* may be regulated by indole or other pathways, not only by *korA* and *korB*.

Indole production of donor cell *SM10*λπ was significantly repressed by CIP in sub-inhibitory concentration. This result was inconsistent with previous report that *E. coli* exhibited greater indole production in the presence of kanamycin which may be isolated from *Streptomyces kanamyceticus* (Umezawa et al., 1957; Han et al., 2011). The reason behind this phenomenon may be that *E. coli* may utilize indole to compete against other microorganisms that could produce antibiotics (Han et al., 2011). However, CIP is a member of synthetic quinolone antibiotics, and indole is reported to stimulate the formation of *E. coli* persisters against quinolone antibiotics (Zarkan et al., 2020). We referred that CIP may in turn play a role through inhibiting indole production.

The selective pressures caused by increases in the use and misuse of antibiotics in medicine and animal feedstuffs account for the spread of antibiotic resistance genes (von Wintersdorff et al., 2016). However, the underlying mechanisms for antibiotic-induced conjugative transfer remain largely unknown. Here, we found that CIP in sub-inhibitory concentration clearly enhanced conjugation frequency in both *SM10*λπ-*PAO1* and *SM10*λπ-*EC600* models. Combining the result that CIP inhibited indole production of donor cell and indole inhibited CIP-induced conjugation frequency, we inferred that indole may play a negative role in CIP-induced conjugation. In our previous work, AHLs secreted from recipient cell *PAO1* worked to inhibit Gm-induced conjugation (Lu et al., 2017a). It seems that a different regulatory mechanism involved in the conjugation process is related to different types of antibiotics and bacteria.

In conclusion, we found that indole may function as a conjugation inhibitor and play a negative role in the process of CIP-induced conjugation. To the best of our knowledge, this is the first report to reveal the regulatory role of indole in conjugation. These results not only enrich our understanding of the biological function of indole but also inspire us to explore a new way to restrain conjugation to further control the spread of antibiotic resistance.

## Materials and Methods

### Bacterial Strains, Plasmids, and Growth Conditions

The bacterial strains and plasmids used in this study are listed in Supplementary Table 2. Some of these strains and plasmids have already been described in previous work (Zeng et al., 2016; Lu et al., 2017b). However, the *tnaA*-deficient strain (Δ*tnaA*-vector) and overexpression of TnaA in wild-type (*SM10*λπ-TnaA) and *tnaA* mutation (Δ*tnaA*-TnaA) strain were developed in this research. Bacteria were grown in a Luria–Bertani (LB) medium or on LB plates containing 1.5% agar unless otherwise indicated. If required, antibiotics were added to LB plates at the following final concentrations: ampicillin (AMP), 100 μg/ml; Gm, 30 μg/ml; rifampin (Rif), 50 μg/ml; and chloramphenicol (C), 16 μg/ml.

### Construction of *E. coli SM10λπ tnaA*-Deficient Mutants and Overexpressed Plasmid

The phage λ Red recombination system was employed for *tnaA* deletion in *E*. *coli SM10*λπ (Datsenko and Wanner, 2000). To construct TnaA overexpression, the *tnaA* gene was cloned into the *Pst*I/*Hin*dIII sites of the pSTV28 vector. More details are provided in the Supplementary Materials and Methods, and corresponding validation can be found in Supplementary Figure 2.

### Growth Curves

The indicated bacterial strains were cultured in LB overnight (8–10 h) at 37°C, then diluted to 1 × 10^7^ CFU/ml (by using the Sysmex UF-1000i automated urine particle analyzer; Tokyo, Japan) with or without treatment, further divided into 12-well plates at a volume of 1 ml per well, and finally grown at 37°C. The samples were collected at the indicated time points from each individual well, and OD600 values were determined.

### Conjugation Experiments

*Escherichia coli SM10λπ* (pUCP24T) worked as donor cells, with the RP4 plasmid integrated in the chromosome, while *PAO1* or *EC600* worked as recipient cells. The pUCP24T plasmid was constructed by inserting the oriT fragment into pUCP24 (West et al., 1994). To achieve transfer, the mobilizable pUCP24T plasmid that contains a gene cassette (*aacC1*) conferring Gm resistance needed to leverage the conjugative apparatus of plasmid RP4 which was integrated in *E. coli SM10λπ*. For mating experiments, equal amounts of donor and recipient cells (1 × 10^7^ CFU/ml, counted by using the Sysmex UF-1000i automated urine particle analyzer; Tokyo, Japan) were mixed in 200 μl LB with or without exogenous indole at 37°C in a 96-well plate. After 6 h of mating, the cultures were vigorously mixed, and 30 μl aliquots of each conjugation mixture were spread on screening agar plates, Gm–ampicillin and Gm–rifampicin plates, since PAO1 is ampicillin resistant and EC600 is rifampicin resistant. The numbers of transconjugant colonies were counted after an overnight incubation at 37°C.

### MIC Determinations

MIC values were determined by the broth microdilution method in poly-styrene microtiter plates (no. 3599; Costar) according to CLSI protocol M07-A8 using cation-adjusted Mueller–Hinton broth (CAMHB) (no. 11865; Oxoid). MICs were interpreted visually after incubating at 37°C for 16 h.

### Indole Assays

The production of extracellular indole was measured by the modified Kawamura–Sato method (Kawamura-Sato et al., 1999; Han et al., 2011). First, Kovac’s reagent (0.4 ml) was added to cultural supernatant (1 ml), followed by sufficient mixing. Then the mixture (0.1 ml) was diluted into an HCl-amyl alcohol solution (0.9 ml, the ratio of HCl and amyl alcohol was 1:3). Finally, OD540 values were determined using synergy H1 microplate reader (BioTek; Winooski, VT, United States), and indole concentration was calculated according to the standard curve.

### Real-Time PCR

Total RNA was extracted using RNAiso Plus reagent (TaKaRa, Dalian, Liaoning, China). Reverse transcription (1 μg of total RNA) was performed with the PrimeScript RT reagent kit (TaKaRa, Dalian, Liaoning, China). The cDNA was subjected to quantitative PCR (qPCR) on a ViiA 7 Dx system (Applied Biosystems, Foster, CA, United States) using SYBR green qPCR master mixes (TaKaRa, Dalian, Liaoning, China). The expression levels of target genes were normalized to that of the internal control gene (*rpoD*) using the 2^–ΔΔ*Ct*^ method. More details about primers are listed in Supplementary Table 3.

### Statistical Analysis

Data are expressed as the means ± standard errors of the means (SEMs) from at least three independent experiments. The differences between groups were analyzed using the Student’s *t*-test when two groups were compared or a one-way analysis of variance (ANOVA) when more than two groups were compared. All analyses were performed using GraphPad Prism, version 5 (GraphPad Software, Inc., San Diego, CA, United States). All statistical tests were two-sided; *P*-values of <0.05 were considered statistically significant.

## Data Availability Statement

All datasets generated for this study are included in the article/Supplementary Material, further inquiries can be directed to the corresponding author/s.

## Author Contributions

RX, YuL, and JP contributed to the conception and design of the study. JL organized the database. JZ performed the statistical analysis. RX wrote the first draft of the manuscript. YuL, JP, JL, and JZ wrote the sections of the manuscript. CC, YaL, and BH contributed to funding acquisition. All authors contributed to manuscript revision, read, and approved the submitted version.

## Conflict of Interest

The authors declare that the research was conducted in the absence of any commercial or financial relationships that could be construed as a potential conflict of interest.
