# Supplementary material for: Indole Inhibits IncP-1 Conjugation System Mainly Through Promoting korA and korB Expression
Source: Front Microbiol. 2021 Mar 19;12:628133. doi: 10.3389/fmicb.2021.628133 (PMC8017341; doi:10.3389/fmicb.2021.628133)
Supplement: Supplementary file 1 [file Presentation_1.pdf]

Supplementary Materials and Methods

Figure S1

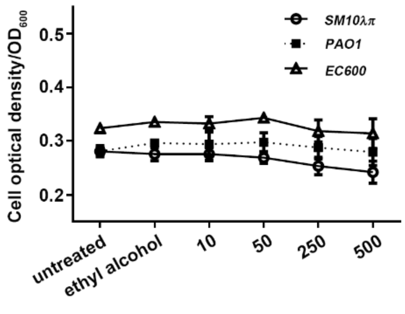

Figure S1. The growth curve of *SM10λπ*, *PAO1* and *EC600* under indoles. Cells (1 × 10^7 CFU/ml) were cultured in the presence of indicated concentrations of indoles and isometric solvent ethyl alcohol at 37°C for 6 h. Values are means ± SEMs from at least three independent experiments.

Figure S2

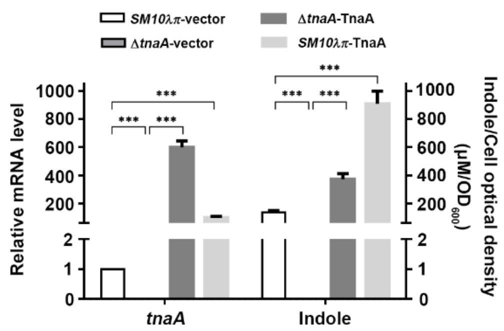

Figure S2. Validation of constructed strains. Indicated strains in the logarithmic phase were collected for extracellular indoles, cell optical density measurement and real-time PCR analysis of *tnaA* expression. The indoles expression level was indicated in terms of extracellular indoles production (μM) divided by cell optical density (OD600). Values are means ± SEMs from at least three independent experiments. ***, $P < 0.001$.

Table S1. Minimal inhibitory concentration (MIC) of *SM10λπ* against ciprofloxacin.

No.	1	2	3	4	5	6	7	8	9	10	11	12
First	-	-	-	-	-	-	-	-	+	+	+	+
Second	-	-	-	-	-	-	-	-	+	+	+	+
Third	-	-	-	-	-	-	-	-	+	+	+	+
CIP (μg/ml)	2^0	2^{-1}	2^{-2}	2^{-3}	2^{-4}	2^{-5}	2^{-6}	2^{-7}	2^{-8}	2^{-9}	2^{-10}	2^{-11}

Table S2. The information of strains and plasmids used in this work.

Strains/plasmids	Genotype or characteristics	Source
Escherichia coli		
SM10$\lambda\pi$	thi thr leu tonA lacY supE recA::RP4-2-Tc::Mu Km λpir	(1)
SM10$\lambda\pi$ Δ*tnaA*	Mutants of *E. coli SM10*$\lambda\pi$ deficient in *tnaA* gene	This work
Δ*tnaA*-vecor	*SM10*$\lambda\pi$ Δ*tnaA* with pSTV28 vector introduced	This work
Δ*tnaA*-TnaA	*SM10*$\lambda\pi$ Δ*tnaA* with pSTV28-TnaA introduced	This work
SM10$\lambda\pi$-TnaA	*E. coli SM10*$\lambda\pi$ with pSTV28-TnaA introduced	This work
EC600	LacZ-, Gm^S, Rif^R	Our lab
Pseudomonas aeruginosa		
PAO1	Wild-type strain, Gm^S, Amp^R	(2)
Plasmids		
pSTV28	Control plasmid, containing P_{lac} promoter, Cm^R	Our lab
pSTV28-*tnaA*	pSTV28 derivative, *Escherichia coli SM10*$\lambda\pi$ *tnaA* overexpression plasmid, controlled by the constitutive P_{lac} promoter, Cm^R	This work
pUCP24T	370 bp oriT fragment from pCVD442 cloned into pUCP24, ori1600, Gm^R	Our lab
pKD3	oriR6K, FRT::cat::FRT template plasmid Cm^R , Amp^R	(3)
pKD46	oriR101 repA101ts P-araB-gam-bet-exo Amp^R	(3)
pCP20	pSC101 temperature-sensitive repliconts, Flp (λ Rp), cI857, Cm^R, Amp^R	(3)

Gm^R, Rif^R, Amp^R and Cm^R stand for gentamycin, rifampin, ampicillin and chloramphenicol resistance, respective.

Table S3. Sequences of RNA and DNA oligonucleotides.

Name	Sense primer (5'-3')	Antisense primer (5'-3')	Source
Promers for qPCR			
tnaA	TCACCCGCGAAACCTACAAA	GTCTTTCATGCACAGCAGGC	Our lab
korA	GCTTACCGAAAGCCAGTTCCAG	GCAAGTTCTTGTCCTCGAACGC	(4)
korB	AAGGAAAAGGGCGCGAAGGAG	TCGATGAGCGCGACCAGTTTC	(4)
*trb*A	TGGAAACTCCCCTACCTCTT	CCACACTGATGCGTTCGTAT	(5)
*trb*B	CGCGGTCGCCATCTTCACG	TGCCCGAGCCAGTACCGCCAATG	(6)
*trf*A	GAAGCCCATCGCCGTCGCCTGTAG	GCCGACGATGACGAACTGGTGTGG	(5)
traI	ATCACGAAGGGAACCATCATC	TTGAACTCTGCTGTGCCGTTGAC	(7)
traJ	CGAACGAAGAGCGATTGAGG	TCGTCGGTGAGCCAGAGTTT	This work
rpoD	TATCTGCTGGAACAGTACGATCGTG	TGTTGTCATCATCGGCGCTG	(8)
Primers fo*r tnaA* promoter cloning			
tnaA	TATGACCATGATTACGAATTTCGGTATAGCAGATG	ACGACGGCCAGTGCCAAGCTACATCCTTATAGCCACTC	This work

Plasmids construction

For construction of TnaA-expressing plasmid, TnaA coding region was amplified by PCR from *E. coli SM10λπ* chromosomal DNA. Amplified fragments were purified by gel extraction kit (ShengGong, Shanghai, China), then digested with *Hind III* and *BamH I* (Takara, Dalian, Liaoning, China), and cloned into the corresponding sites of pET32a (+).

Construction of *E. coli SM10λπ tnaA* deficient mutants

Construction of *E. coli SM10λπ tnaA* mutant followed the same steps described by Datsenko *et al* (3) except 100 mM L-arabinose was used to induce P_{araB} promoter of pKD46. Briefly, the insert fragments were amplified with primer D-*tnaA*-F (TGTAATATTCACAGGGATCACTGTAATTAAAATAAATGAAGGATTATGTATG TAGGCTGGAGCTGCTTCG) and D-*tnaA*-R (TGTAGGGTAAGAGAGTGGCTAACATCCTTATAGCCACTCTGTAGTATTAAAT GGGAATTAGCCATGGTCC). Afterwards, 100 μL cells were transfected with 1000ng PCR products using electroporation according to the manufacturer's instructions. Recombinants were selected on LB agar (Cm, 15μg/mL) and then transformed with pCP20 to eliminate FRT-flanked *cat* gene. Both mutants were verified by PCR (primers C-*tnaA*-F: TTTGCCCTTCTGTAGCCATC and C-*tnaA*-R: ACCATAACACCCCAAAATGC) and DNA sequencing.

1. Simon R, Priefer U, Pühler A. A Broad Host Range Mobilization System for *In Vivo* Genetic Engineering: Transposon Mutagenesis in Gram Negative Bacteria. *Bio/Technology* (1983) 1:784-91.

2. Stover CK, Pham XQ, Erwin AL, Mizoguchi SD, Warrener P, Hickey MJ, et al. Complete genome sequence of *Pseudomonas aeruginosa PAO1*, an opportunistic pathogen. *Nature* (2000) 406(6799):959-64.

3. Datsenko KA, Wanner BL. One-step inactivation of chromosomal genes in *Escherichia coli* K-12 using PCR products. *Proc Natl Acad Sci U S A* (2000) 97(12):6640-5.

4. Zatyka M, Bingle L, Jones AC, Thomas CM. Cooperativity between KorB and TrbA repressors of broad-host-range plasmid RK2. *J Bacteriol* (2001) 183(3):1022-31.

5. Qiu Z, Yu Y, Chen Z, Jin M, Yang D, Zhao Z, et al. Nanoalumina promotes the horizontal transfer of multiresistance genes mediated by plasmids across genera. *Proc Natl Acad Sci U S A* (2012) 109(13):4944-9.

6. Wang Q, Mao D, Luo Y. Ionic Liquid Facilitates the Conjugative Transfer of Antibiotic Resistance Genes Mediated by Plasmid RP4. *Environ Sci Technol* (2015) 49(14):8731-40.

7. Balzer D, Pansegrau W, Lanka E. Essential motifs of relaxase (TraI) and TraG proteins involved in conjugative transfer of plasmid RP4. *J Bacteriol* (1994) 176(14):4285-95.

8. Savli H, Karadenizli A, Kolayli F, Gundes S, Ozbek U, Vahaboglu H. Expression stability of six housekeeping genes: A proposal for resistance gene quantification studies of *Pseudomonas aeruginosa* by real-time quantitative RT-PCR. *J Med Microbiol* (2003) 52(Pt 5):403-8.
